# Implantation of undifferentiated and pre-differentiated human neural stem cells in the R6/2 transgenic mouse model of Huntington’s disease

**DOI:** 10.1186/1471-2202-13-97

**Published:** 2012-08-09

**Authors:** Gehan El-Akabawy, Ivan Rattray, Saga M Johansson, Richard Gale, Gillian Bates, Michel Modo

**Affiliations:** 1 Department of Neuroscience, King’s College London, Institute of Psychiatry, London, SE5 9NU, United Kingdom; 2 Menoufia University, Faculty of Medicine, Menoufia, Egypt; 3 Department of Medical and Molecular Genetics, Guy’s Hospital, King’s College London School of Medicine, London, SE1 9RT, United Kingdom; 4 Department of Radiology, McGowan Institute for Regenerative Medicine, University of Pittsburgh, 3025 East Carson St, Pittsburgh, PA15203, USA

**Keywords:** Neural stem cells, Human, Cell therapy, R6/2, Pre-differentiated cells, DARPP-32, Striatum, Purmorphamine, Huntington’s disease, Behaviour, MRI, Cell survival

## Abstract

**Background:**

Cell therapy is a potential therapeutic approach for several neurodegenetative disease, including Huntington Disease (HD). To evaluate the putative efficacy of cell therapy in HD, most studies have used excitotoxic animal models with only a few studies having been conducted in genetic animal models. Genetically modified animals should provide a more accurate representation of human HD, as they emulate the genetic basis of its etiology.

**Results:**

In this study, we aimed to assess the therapeutic potential of a human striatal neural stem cell line (STROC05) implanted in the R6/2 transgenic mouse model of HD. As DARPP-32 GABAergic output neurons are predominately lost in HD, STROC05 cells were also pre-differentiated using purmorphamine, a hedgehog agonist, to yield a greater number of DARPP-32 cells. A bilateral injection of 4.5x10^5^ cells of either undifferentiated or pre-differentiated DARPP-32 cells, however, did not affect outcome compared to a vehicle control injection. Both survival and neuronal differentiation remained poor with a mean of only 161 and 81 cells surviving in the undifferentiated and differentiated conditions respectively. Only a few cells expressed the neuronal marker Fox3.

**Conclusions:**

Although the rapid brain atrophy and short life-span of the R6/2 model constitute adverse conditions to detect potentially delayed treatment effects, significant technical hurdles, such as poor cell survival and differentiation, were also sub-optimal. Further consideration of these aspects is therefore needed in more enduring transgenic HD models to provide a definite assessment of this cell line’s therapeutic relevance. However, a combination of treatments is likely needed to affect outcome in transgenic models of HD.

## Background

Despite very significant advances in understanding the causes of Huntington’s disease (HD), an efficacious therapy remains elusive [1]. Cell therapy is a putative treatment for HD that could slow down neurodegeneration, replace lost cells and potentially provide a long-term benefit. Preclinical and proof-of-principle clinical trials using fetal tissue grafts suggest that therapeutic benefits are possible [2-4]. However, the usage of human fetal tissue grafts raises several ethical, logistical, and safety concerns. Notably, the procurement of large quantities of human fetal tissue at an appropriate developmental stage from elective abortions, establishing the absence of genetic disease or any other potentially harmful contaminations, as well as the heterogeneous (multiple donors) nature of the grafts, limit their potential usage in a routine clinical setting [5].

During the last decade, neural stem cell lines emerged as a potential alternative to fetal tissue grafts, as they can be maintained and expanded in vitro. Human neural stem cells (hNSCs) afford a sustainable and scalable homogenous cell source to treat large cohorts of patients [6]. There is evidence that cell therapy can slow down neurodegeneration and ameliorate behaviour in rat models of Huntington’s disease [2,7-9]. Replacement of lost cells is, however, a greater challenge. Although human neural stem cells can differentiate into neurons after implantation [10,11], improvements of functional deficits by fetal striatal transplants into a lesioned rat striatum is associated with DARPP-32 neurons within the transplants [12-14]. Despite good survival and differentiation of human neurons in the rats, differentiation of cells into DARPP-32 neurons remains a challenge [10,15]. Pre-differentiation of cells prior to implantation into a DARPP-32 phenotype therefore could potentially result in an improved outcome [16]. Proof-of-principle of this strategy for mouse embryonic and neural stem cells have previously been demonstrated in rat or mouse excitotoxic models of HD [9,17].

However, to successfully progress this approach to a routine clinical application, it is essential to develop this approach for human stem cells [18]. hNSC lines, such as the STROC05 cell line (derived from the ganglionic eminence of a 12 week-old fetus) have the potential to differentiate in vitro into DARPP-32 cells [19,20] and potentially could provide a source of pre-differentiated DARPP-32 neurons for implantation. Ideally the potential efficacy of either undifferentiated or pre-differentiated cells is evaluated in a genetic model that exhibits a progressive phenotype resembling that of human HD. One of these models, the R6/2 transgenic mouse model (expresses the exon1 of the human HD gene), is most commonly used to screen new therapies for Huntington’s disease [21]. The impact of undifferentiated and pre-differentiated STROC05 cells on behavioural impairments and brain atrophy was therefore evaluated in the R6/2 mouse model of Huntington’s disease.

## Methods

### Human neural stem cell line (STROC05)

The cmyc-ER^TAM^ conditionally immortalized human striatal neural stem cell line (STROC05, kindly provided by ReNeuron Ltd., Surrey, UK) was previously described [19]. In brief, STROC05 cells were isolated from the whole ganglionic eminence of 12-weeks-old human fetal brain. The cmyc-ER^TAM^ gene was transfected into cells with the retroviral vector pLNCX-2 (Clontech). Transfected cell colonies were isolated following neomycin selection before being expanded into a clonal cell line [22]. To maintain proliferation through the conditional immortalization gene, 4-hydroxy-tamoxifen (4-OHT, 100 nM/ml; Sigma-Aldrich, UK) was added to proliferation media. The STROC05 cell line was expanded in T75 tissue culture flasks (Falcon, UK). Flasks were coated with mouse laminin at a concentration of 1:100 (mouse, 10 μg/ml; Trevigen, USA) for at least 2 hours at 37°C. Medium was changed every 2 days and cells were passaged at 90% confluence. The expansion media consisted of Dulbecco’s Modified Eagle’s Medium/Ham’s F12 (DMEM:F12; Gibco, UK) which was supplemented with additional components (Table 1). To stimulate proliferation, growth factors, such as basic fibroblast growth factor-2 (bFGF-2, 10 ng/ml; Peprotech, UK) and epidermal growth factor (EGF, 20 ng/ml; Peprotech, UK), were added to the media. 

**Table 1:** Composition of cell culture media to expand the STROC05 cell line

**Component**	**Concentration**	**Supplier**
**DMEM: F:12**	**Base media**	**Gibco**
Human albumin solution	0.03%	Baxter
Human insulin	5 μg/ml	Sigma
L-glutamine	2 mM	Sigma
Putrescine DiHCl	16.2 μg/ml	Sigma
Sodium Selenite	40 ng/ml	Sigma
L-Thyroxine (T4)	400 ng/ml	Sigma
Tri-iodo-thyronine (T3)	337 ng/ml	Sigma
Progesterone	60 ng/ml	Sigma
Corticosterone	20 μg/ml	Sigma
*bFGF-2	10 μg/ml	PeproTch
*EGF	10 μg/ml	Invitrogen
*4-OHT-tamoxifen	100nM/ml	Sigma

Factors with a * were removed to induce a spontaneous differentiation of cells.

### In vitro differentiation of STROC05 cells

To induce neuronal differentiation and increase the proportion of DARPP-32 cells, STROC05 cells were grown *in vitro* for 21 days on laminin (mouse, 10 μg/ml, Trevigen) and poly-l-lysine (PLL, 100 μg/ml, Sigma) coated T175 flasks with 90% confluence, as previously described [20]. For the first week, differentiation was induced using media that contained all components from the proliferation media, with the exception of bFGF-2, EGF and 6-OHT. For the 2^nd^ and 3^rd^ week of differentiation, media consisted of neurobasal media (Gibco) supplemented with B-27 (Gibco), L-Glutamate (Sigma) and Purmorphamine. For the 2^nd^ week of differentiation, bFGF was added again to the media as a survival factor [23] and to promote a rostral positional specification of neurons [24,25], but was omitted again for the 3^rd^ week of differentiation as positional specification in most cells is completed. Purmorphamine (1 μM, Calbiochem) was added to the culture media throughout the 3 weeks of differentiation.

### Effect of harvesting on cell viability and differentiation

As differentiated cells are very vulnerable when removed from tissue culture flasks, it is essential to establish whether harvesting these cells after long-term differentiation affects their viability and differentiation status. For this, cells were harvested with Trypzean EDTA for less than five minutes at 37°C, followed by adding a soybean trypsin inhibitor to inactivate the enzymatic activity. After harvesting, cells were centrifuged for 5 minutes at 1500 rpm and the cell pellet was re-suspended in 1 ml of DMEM. Using the trypan blue exclusion test, cells were counted and viability was established to be 89.5%. Cells were re-seeded on laminin-coated cover slips in 24 well plates at 100,000 cells per well. After 24 h, viability of these re-seeded conditions was evaluated again using the live/dead stain (viability/cytotoxicity kit for mammalian cells, Gibco) and compared to cells that were not harvested. For the live/dead stain, media was aspirated and cells were washed once with PBS prior to incubation with 2 μM calceinAM (to detect live cells) and 4 μM ethidium homodimer-1 (EthD-1) (to detect dead cells) in PBS (500 μL per well) for 45 minutes at 37°C. Photos were taken immediately using a fluorescent microscope (Zeiss). A separate set of coverslips were fixed with 4% Parafix (Pioneer) for 5 min. Immunohistochemistry was used to establish if harvesting of cells would affect the proportion of neurons (1:500, mouse anti-β-III-tubulin, Tuj, AB7751, Abcam) and specifically DARPP-32 neurons (1:500, rabbit anti-DARPP-32, AB1656, Chemicon) within the cell suspension. After overnight incubation (at room temperature) with the primary antibody, an appropriate secondary ALEXA594 (1:1000, Molecular Probes) was applied for 60 min prior to attaching the coverslips to microscopic slides with Vectashield for fluorescence containing DAPI (Vector Laboratories). Total DAPI, as well as Tuj and DARPP-32 cells, were counted under a Zeiss Axioscope.

### R6/2 mice

All procedures of this study were carried out according to the UK Animals (Scientific procedures) Act 1986 (PPL70/6445), as well as the ethical review process of King’s College London. A widely used and well characterized mouse transgenic model of Huntington’s disease, R6/2 mice present with a rapid disease onset that is evident as early as 6 weeks of age. Especially the development of a clear behavioural phenotype in the R6/2 compared to the N171-82Q or HDH^111^ is important to establish a potential therapeutic efficacy.

The average life span of R6/2 mice with 210 CAG repeats is approximately 16 weeks of age [26]. Here, R6/2 mice were generated from a colony that was maintained by backcrossing R6/2 males to (CBA × C57BL/6) F1 females (B6CBAF1/OlaHsd, Harlan, UK). Mice were kept in standard housing conditions, on a standard chow diet with water available *ad libitum*. During the last 2 weeks of the study (12 and 13 weeks of age), a mash diet was prepared by soaking chow pellets in water. These were placed in the floor of the cages within easy reach of the motor impaired R6/2 mice. Transgenic mice were identified by Polymerase Chain Reaction (PCR) on an ear tissue sample at 4 weeks of age, as previously described [27].

Forty female mice were randomized into 4 groups; wild type mice receiving vehicle (WT-veh, n = 10), R6/2 mice receiving vehicle (R6/2-veh, n = 10), R6/2 mice receiving undifferentiated cells (R6/2-undiff, n = 10), and R6/2 mice receiving long-term purmorphamine-differentiated cells STROC05 (R6/2-diff, n = 10). Mice were group-housed 4 per cage containing mixed genotypes (one from each experimental group) to ensure comparable standard housing conditions, as described by Hockley et al. [26].

### Cell implantation

On the day of transplantation, the cells were harvested by incubation with Trypzean EDTA for less than five minutes at 37°C, followed by adding soybean trypsin inhibitor to inactivate the enzymatic activity. After harvesting, cells were centrifuged for 5 minutes at 1500 rpm and the cell pellet was re-suspended in 2 ml of DMEM for cell counting. Cells were suspended in vehicle consisting of 2.5 ml of DMEM and 3.75 ml of Hypothermosol (BioLife Solutions) at a concentration of 7.5 × 10^4^ cells/μl. Using the trypan blue exclusion test, viability was determined to be 89%.

At 7 weeks of age, mice underwent stereotactic surgery for the injection of NSCs. This allowed sufficient time to conduct pre-implantation MRI scans, as well as behavioural test, after animals were weaned at 4 weeks of age from their mothers. Additionally, animals’ genotype was determined and animals were randomly allocated to their experimental groups based on a sequence of random numbers. Although at 7 weeks of age, R6/2 mice do not exhibit a motor deficit [28], they do nevertheless already show signs of brain atrophy [29]. Impor-tantly, R6/2 mice do not display any neuronal loss [28]. At this age, there is also a decrease, as well as morphological abnormalities, in microglia [30].

For cell implantation, anaesthesia was induced through isoflurane inhalation (Abbott) at 4-5%, then maintained at 1.5-2%. Animals were mounted in a stereotaxic frame and a sagittal incision was carefully made followed by the drilling of two burr holes. Either 6 μl (3 μl per side, 0.5 μl/min) of cells or vehicle were injected with a 22 Gauge needle attached to 10 μl Hamilton syringe using a convection-enhanced delivery [31] at Anterior-Posterior +0.5 mm (in relation to Bregma), Lateral ±2 mm and −3.5 mm below the surface of the dura. The deposit was divided into two equal amounts; one was injected at −3 mm (after retraction of the needle by 0.5 mm) and the other at −2.5 mm. After injection, the syringe was left in place for 5 minutes and slowly withdrawn over 3 minutes, followed by suturing of the incision. During the surgery, body temperature was controlled using a homeostatic heating pad set at 37°C. No immunosuppression was given as STROC05 cells exhibit a robust survival in the 3-nitropropionic acid rat model of Huntington’s disease over 90 days (Additional file 1: Figure S1), as well as wild-type mice (Additional file 2: Figure S2).

After surgery, post-operative care included fluid-replacement (0.1 ml saline/animal) and a local analgesic (EMLA cream 5%; AstraZeneca, UK). The animals were singly caged with softened food pellets and water available *ad libitum* for 24 h before being returned to their home cages.

### Body weight

Weight loss is a prominent symptom in R6/2 mice [28,32]. Body weight has often been used as a reliable outcome measure to assess the beneficial effect of different therapeutic approaches in R6/2 mice [26,33-37]. Mice were weighted weekly from the time of weaning (4 weeks) until the end of the study. To avoid the impact of diurnal feeding habits, body weight was obtained weekly on the same day and time.

### Behavioural battery

For each behavioural test, the running order of animals was based on a randomization of the cages, but within each cage (containing WT and R6/2), mice were run sequentially. Animals within each cage were randomly chosen for each trial. If more than one trial was conducted, this was run in the same sequence.

#### Rotarod

The rotarod is considered a very sensitive and reliable motor task to assess motor coordination in HD transgenic mice [26,38]. R6/2 mice are known to have impaired rotarod performance [39,40]. According to a standard protocol [26], mice were placed on a rotarod (Ugo Basile) with a 3 cm diameter rod at a constant speed of 4 rpm for 20 sec. After this acclimatisation period, the rod speed accelerated from 4 to 40 rpm over 300 sec. Latency for mice to fall from the rod was recorded. Rotarod performance was assessed over three successive days with 3 trials per day. The first assessment day was always excluded from analysis. Mice were tested one week pre-transplantation, as well as at 1, 3, and 5 weeks post-transplantation.

#### Open field

The open field test has been used extensively as a reliable measure to evaluate locomotor activity and anxiety-like behaviour in R6/2 mice [41,42]. A custom-built 100 cm diameter and 35 cm deep circular open field arena (Engineering & Design Plastics) was divided into outer and inner zones by a circle drawn 4 cm from the outer walls. Mice were placed individually in the outer zone facing the centre of the maze with their behaviour being automatically recorded by a camera for a period of 5 min. Data was subsequently analysed using Ethovision XT7.0 software (Noldus). The arena was cleaned between mice to prevent behavioural influences from the odours of previous trials. Total distance travelled (locomotion) and time spent in the outer zone (thigmotaxis, indicative of anxiety-like behaviour) were measured one week pre-transplantation, as well as at 1, 3, and 5 weeks post-transplantation.

#### Grip strength

Grip strength analysis is a reliable and sensitive test to evaluate muscular strength in R6/2 mice [26,39,42]. To measure forelimb grip strength, mice were lowered towards the grid to grab it with both front paws. Mice were gently pulled back until they released their grip and the equipment automatically measured the force required to pry the mouse from the grid. A single session consisting of 5 consecutive trials was recorded once a week at 4, 5, and 6 weeks post-grafting. As low scores may be due to the mouse failing to grip the grid effectively, the best three scores of the five trials were averaged.

### Magnetic Resonance Imaging (MRI)

Six weeks following cell implantation, mice were anesthetised using isoflurane (4-5% induction, 1.5-2% maintenance in 0.7 l/min medical air and 0.3 l/min oxygen) and fixed within a head holder/respiration mask to reduce head movement. MR images were acquired using a 7 Tesla magnet (Varian), equipped with a 100 Gauss gradient set and a 39 mm transmission/receive coil (Rapid). A T_2_-weighted multi-echo multi-slice (MEMS) sequence was used (TR = 2500 ms, minimum TE = 10 ms, number of echo = 8, echo spacing = 10 ms, averages = 4, matrix = 128x128, and FOV = 20 × 20 mm). Thirty coronal slices with 0.5 mm thickness were acquired across the mouse brain. Manual segmentation of anatomical regions of interest (ROIs, Additional file 3: Figure S3), including whole brain, striatum, cortex, hippocampus, and lateral ventricle, was performed using JIM 5.0 (Xinapse). Criteria used to define ROIs are summarized in Table 2. Manual segmentation of the same structure at two separate occasions yielded an intra-rater discrepancy of less than 2% error.

**Table 2:** Anatomical criteria adopted to manually segment ROIs

**Brain regions**	**Anatomical boundaries for regions of interest (ROIs)**
Whole brain	Anterior - the first slide behind the eye sacs
	Posterior - the first slide where the hemispheres disappear
Lateral ventricle	Hyperintense T_2_ signal of the cerebrospinal fluid
Striatum	Dorsal - corpus callosum
	Lateral - external capsule
	Medial - lateral ventricle
	Ventral - anterior commissure
Cortex	Internal - corpus callosum & vertical line from lateral edge
	External - skull
	Inferior - horizontal line from anterior commissure
Hippocampus	External - corpus callosum
	Internal - external capsule

### Immunohistochemistry

After MRI scanning, anesthetized animals received an intracardial perfusion of saline followed by 4% Parafix (Pioneer). Brains were excised and post-fixed for 24 h at 6°C before being cryoprotected in 30% sucrose at 6°C. Sections (40 μm) were cut on a freezing sliding microtome in the coronal plane and stored at −20°C in tissue cryoprotective solution (25% glycerine, 30% ethylene glycol, and 50% PBS).

To identify transplanted cells, sections were stained with a mouse anti-human nuclear protein (HNA) antibody (1:400, MAB1218, Millipore). For this, sections were rinsed with PBS, blocked for 30 minutes in 0.1% H_2_0_2_ as inhibitor for endogenous peroxidase activity (Sigma), followed by 60 min incubation in 10% blocking solution (10% normal goat serum in 0.3% Triton X-100 PBS) at room temperature (RT, 21°C). To block the non-specific binding of endogenous biotin, the sections were incubated with avidin-biotin blocking solutions (Vector) for 30 min. The sections were incubated with the HNA antibody at RT for an hour, followed by 10 min of incubation at RT with secondary biotinylated anti-mouse antibody (1:200, Vector), and 5 min at RT with an avidin-biotinylated-peroxidase complex (1:100 in PBS, Vector). Secondary antibody binding was visualized using 3,3’-diaminobenzoic acid (DAB, Sigma) dissolved in PBS with the addition of H_2_0_2_ to a concentration of 0.03% immediately before use. Finally, the sections were washed in PBS, mounted onto glass slides, dehydrated for 5 min in each of 70, 85, 90, and 100% alcohol, cleared by xylene, and coverslipped with Entellen (Merck, UK).

For fluorescence immunohistochemistry, sections were incubated for 60 min in 10% blocking solution (10% normal goat serum in 0.3% Triton X-100 in PBS) at RT, followed by 30 min of an avidin-biotin blocking solution (Vector). Sections were then incubated with appropriate primary antibodies against transplanted cells (mouse anti-HNA 1:400), neurons (rabbit anti-Fox3, 1:500, ab104225, Abcam), or DARPP-32 neurons (rabbit anti-DARPP-32, 1:500). After overnight incubation with the primaries, an appropriate secondary antibody (1:200, ALEXA 350; 1:500, ALEXA 647, Molecular Probes) was applied for 60 minutes at RT. Sections were rinsed in PBS and mounted in Vectashield with DAPI.

### Statistical analyses

Statistical analyses were conducted using GraphPad Prism 5.03 (GraphPad Software, San Diego, California, USA) to determine significant differences (p < .05) between *in vitro* and post-mortem immunohistochemistry (independent samples *t*-test), as well *in vivo* measures (repeated-measures two-way ANOVA). Bonferroni post-hoc tests were applied if ANOVAs revealed a significant result. All error bars on graphs are displayed as the standard error of the mean (SEM).

## Results

### Differentiated cells retain viability and DARPP-32 phenotype after harvesting and re-seeding

**Figure 1:**
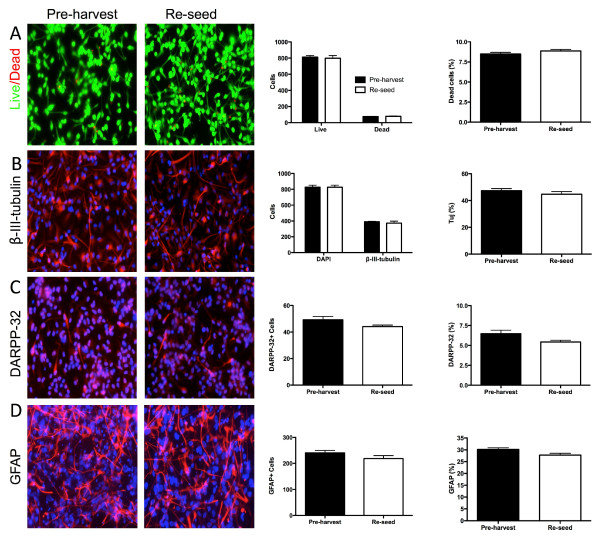
**Viability and neuronal phenotype of re-seeded cultures.** Harvesting and re-seeding of long-term differentiated cultures did not significantly affect their viability (**A**, Live/Dead stain), neuronal differentiation (**B**,β-III-tubulin, Tuj), DARPP-32 (**C**), or astrocytic differentiation (**D**).

To assess whether transplantation of long-term differentiated cells is possible, long-term differentiated cultures were harvested and reseeded to measure potential effects on viability and neuronal differentiation. Viability straight after harvesting of differentiated cells was above 90% as indicated by the trypan blue exclusion test. This good viability was maintained after re-seeding these cells for 24 h (Figure 1A). The harvesting re-seeding procedure also did not reduce the neuronal population (Figure 1B). The number of β–III-tubulin- and DARPP-32-positive cells remained fairly consistent (Figure 1C). The number and percentage of astrocytes also was consistent between pre-harvesting conditions and re-seeding (Figure 1D). These results suggest that the harvesting re-seeding process did not significantly affect the viability of differentiated cells and the neuronal population is very similar to the pre-harvest condition.

### Cell implants do not impact on weight loss

**Figure 2:**
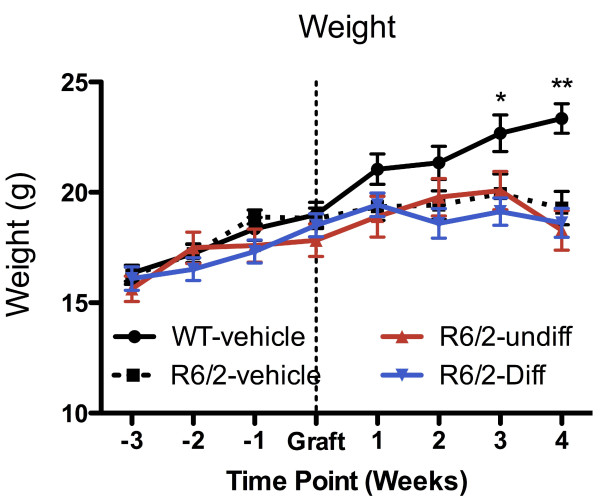
**Body weight.** Weight gain between groups was equivalent up to 7 weeks of age when animals were grafted. Post-implantation WT mice with vehicle injection continued to gain weight. All R6/2 mice started to lose weight 3 weeks post-grafting (11 weeks of age). There was no effect of the implantation of undifferentiated or differentiated cells on body weight.

Body weight is a reliable indicator of the overall health of R6/2 mice. The body weight of wild type (WT) and R6/2 mice (n = 10/genotype) steadily increased until 8 weeks of age (1 week post-implantation, Figure 2), after which they cease to gain weight. By 3 weeks post-implantation, R6/2 mice had significantly lower body weight compared to WT mice. Animals that received undifferentiated or differentiated cells followed the same weight pattern than those R6/2 mice that received a vehicle injection. These results suggest that cell implantation did not impact on weight loss in R6/2 mice.

### Neither undifferentiated, nor differentiated cells improve behavioural deficits

**Figure 3:**
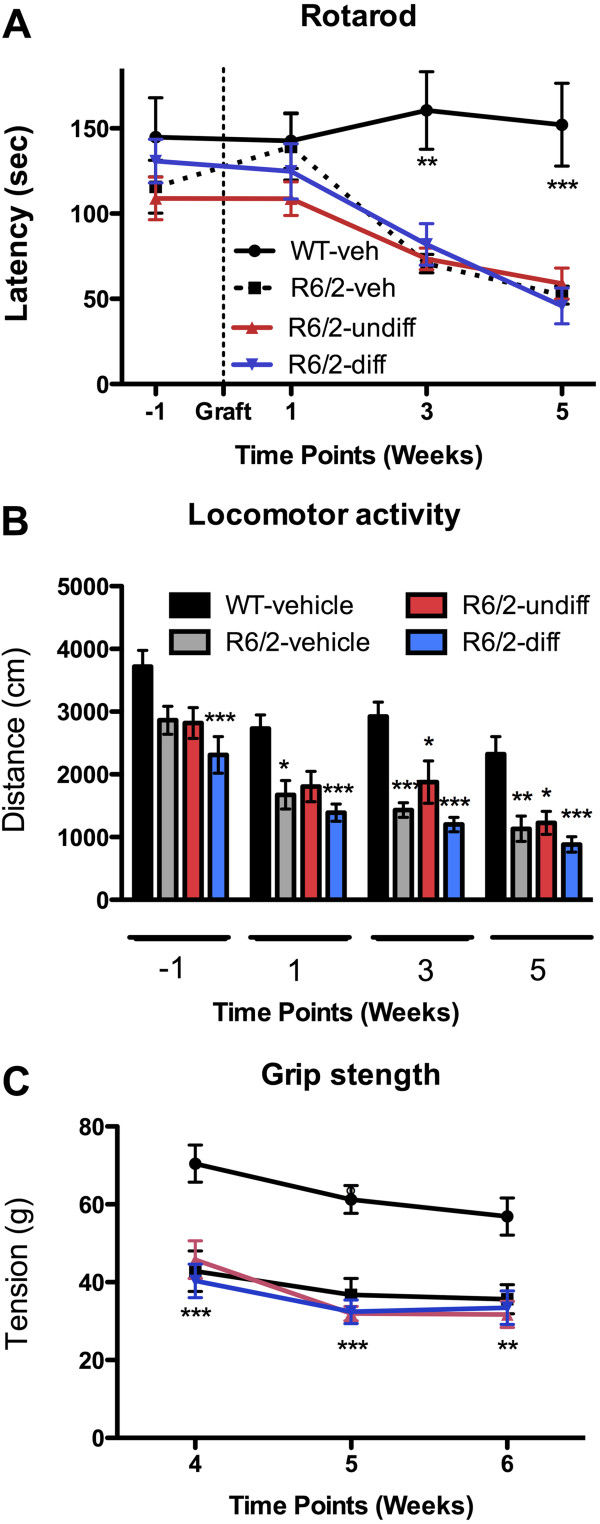
**Behaviour.A.** Rotarod: no effect of genotype on rotarod performance was detected one week pre- or post-grafting. However, a significant impairment was evident in R6/2 mice at 3 and 5 weeks post-grafting which was not improved through the implantation of undifferentiated or differentiated cells. **B.** Open field: the total exploratory activity of R6/2 was reduced compared to WT controls at all time points tested. Neither undifferentiated, nor differentiated, cells attenuated the deterioration of R6/2 exploratory behaviour. **C.** Grip strength: all R6/2 mice showed significantly impaired performance compared to WT mice at all time points. No beneficial effect of treatment was evident. (* p < 0.05, **p < 0.01, and ***p < 0.001).

The development of a progressive behavioural phenotype is a key characteristic of R6/2 mice. Up to 8 weeks of age (1 week post-implantation), the R6/2 animals performed as well as WT controls on the rotarod (Figure 3A), but gradually thereafter their rotarod performance deteriorated as compared to WT controls. Implantation of cells (undifferentiated or differentiated) did not prevent this deterioration. A significant locomotor deficit was already evident in R6/2 animals pre-implanted at 7 weeks of age (Figure 3B). This deficit gradually worsened and the cell therapy had no significant impact. There was also no significant alteration in anxiety-like thigmotaxis behaviour in the R6/2 mice (data not shown). Grip strength was consistently impaired in the animals between 4 and 6 weeks post-grafting, and no improvement due to cell implantation was evident (Figure 3C). Therefore, neither the bilateral implantation of undifferentiated, nor differentiated cells significantly impacted on the emergence or the progression of clear behavioural deficits in the R6/2 mouse model of Huntington’s disease.

### Cell implants did not reduce brain atrophy

**Figure 4:**
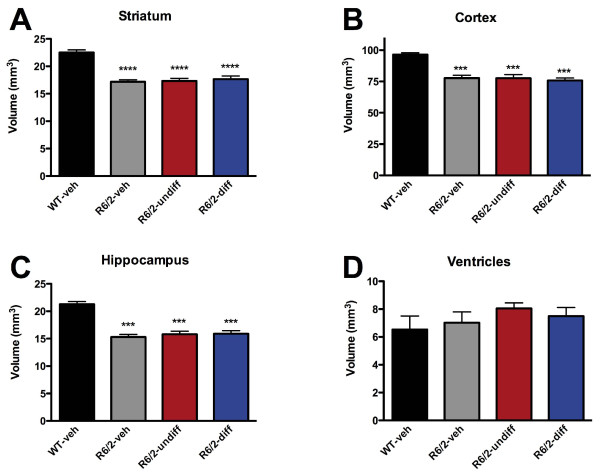
**MRI.** Volumetric analysis revealed a significant atrophy in striatal (**A**), cortical (**B**), and hippocampal tissue (**C**). Lateral ventricles (**D**) were minimally enlarged, but no significant statistical effect was evident. There was no treatment effect of the implantation of either undifferentiated or differentiated cells. (* p < 0.05, **p < 0.01, and ***p < 0.001).

To determine whether cell implants reduced the brain atrophy of R6/2 mice compared to WT controls, T_2_-weighted MRI scans were acquired 6 weeks post-grafting (13 weeks of age). R6/2 mice injected with vehicle, undifferentiated or differentiated exhibited similar levels of atrophy in the striatum (Figure 4A), cortex (Figure 4B) and hippocampus (Figure 4C). Ventricular volume was comparable to WT animals (Figure 4D). Therefore there was no evidence to support a neuroprotective effect of cell implantation.

### Survival and differentiation of cell implants

**Figure 5:**
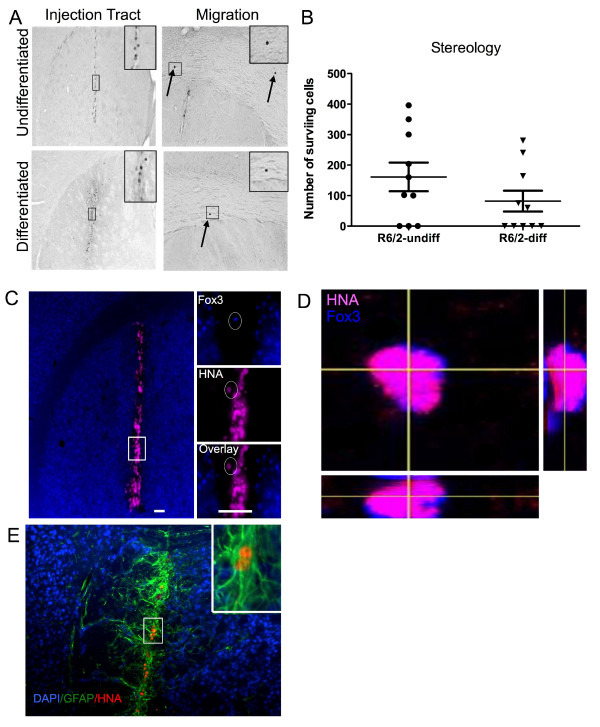
**Survival of the transplanted cells.** A small population of implanted cells survived (**A**). Human cells (human nuclear antigen + cells in pink) were mostly found within the injection tract in the striatum. A select few individual cells were observed migrating along the corpus callosum. Stereological cell counts revealed no significant difference between undifferentiated and differentiated cell implantation (**B**). However, cell survival was very variable with some animals having no surviving cells. Neuronal differentiation, as determined by FOX3 staining, of implanted cells was very poor (**C**&**D**). Most implanted cells differentiated into astrocytes (**E**), whereas others did neither express markers of neurons nor astrocytes. (Scale bar 200 μM).

The survival and differentiation of the implanted cells are essential to guarantee a potentially beneficial effect. Post-mortem immunohistochemical analyses 6 weeks post-implantation indicated that in 70% of R6/2-undiff and 50% of R6/2-diff animals some STROC05 cells survived six weeks post-implantation. A re-analysis of the behaviour and MRI results indicated that exclusion of animals without surviving cells did not significantly affect outcome (Additional file 4: Figure S4). Surviving cells were mostly confined to the injection tract (Figure 5A), with a select few showing a limited migration in the corpus callosum. In the left hemisphere of R6/2-undiff mice, only 161.2 ± 46.8 STROC05 cells survived, whereas 81.9 ± 34.16 cells survived in R6/2-diff animals (Figure 5B). However, given the wide variability within each group, a statistically significant difference between these two types of implants in terms of cell survival could not be detected. Despite the generally poor cell survival, a small number of STROC05 cells expressed Fox3 in both the R/6-undiff (1.2%) and R6/2-diff (2%) conditions (Figure 5C&D). DARPP-32 was not detected in any of the implanted cells. Almost all implanted cells were GFAP-positive (Figure 5E) suggesting that predominantly astrocytic cells survived, whereas neurons did not. Therefore, most implanted cells did not survive by 6 weeks post-implantation and pre-differentiation of STROC05 cells did not increase the presence of neuronal cells post-grafting.

## Discussion

Cell therapy for Huntington’s disease is potentially an important intervention to delay, stabilize and/or improve impairments. These therapeutic effects are well documented in animal models, but more limited, albeit positive, evidence is available in patients with Huntington’s disease that received fetal tissue transplants [43]. However, in the present study, the STROC05 human neural stem cell line in the R6/2 mouse model of HD did not promote recovery. It is important to recognize that a multitude of requirements need to be met for this therapy to be successful and several explanations need to be considered to account for our results: 1) STROC05 cells are not efficacious in HD, 2) insufficient cells survived to promote recovery, 3) there was an insufficient neuronal/DARPP-32 differentiation of cells, and 4) it is also conceivable that the R6/2 model might be too aggressive to evaluate hNSC as a restorative treatment.

### Lack of efficacy and poor cell survival

Therapeutic efficacy in Huntington’s disease is considered to be associated with a decrease in neurodegeneration, as well as a replacement of lost striatal DARPP-32+ GABAergic output neurons. An intra-striatal injection of fetal-derived neural progenitors/stem cells [2,13], NSC lines [7,8], as well as mesenchymal cells [44] produces an improvement in behavioural impairment. Even an intravenous injection of mesenchymal cells can achieve improvements in Huntington’s disease with only a small fraction of cells penetrating the brain [45]. However, human neural stem cells from the STROC05 neural stem cell line did not improve outcome in the R6/2 mouse model of HD.

It is therefore important to consider why STROC05 cells did not improve outcome. Foremost of all, survival of cells after implantation was rather poor with only 161 human cells surviving in one hemisphere. Although there have been reports of behavioural changes with 124 cells surviving in stroke [46], most efficacious studies using cell implantation in Huntington’s disease report survival rates of 2 × 10^4^ cells [2]. Interestingly, STROC05 survival in the 3NPA rat model of HD resulted in 2.5 × 10^4^ cells surviving at 3 months. It is therefore conceivable that either the progressive pathology or the mouse host are factors that affect the long-term survival of these cells. Improving cell survival in a mouse host will be key to establishing whether the poor cell survival is the reason for the lack of efficacy. While there was no evidence here of graft rejection, it is conceivable that an early immune response could have affected cell survival and hence efficacy. If this were the case, administration of immunosuppressants and anti-inflammatory treatment would be expected to improve graft survival and potentially provide sufficient cell survival to promote recovery.

Although the survival of cells is thought to be essential to establish recovery by means of intracerebral hNSC implantation, the lack of differentiation of STROC05 cells might also preclude recovery. Especially, the differentiation of cells into striatal DARPP-32+ GABAergic output neurons has been considered to be directly linked to the degree of functional recovery [47,48]. One approach to increase the number of DARPP-32+ neurons from implanted cells is to direct their differentiation prior to injection. This can either be achieved using chemical factors or genetic engineering [9,18,20,49]. Although the hedgehog agonist purmorphamine here increased the differentiation of STROC05 cells into DARPP-32+ neurons over 3 weeks *in vitro* without affecting viability when these cells are re-suspended, none of the cells had survived for 6 weeks post-implantation. It is conceivable that this is a reflection of the overall poor survival of cells, but it would be reasonable to expect that some improvement in neu-ronal survival of implanted cells could be expected after implantation of pre-differentiated cells. Nevertheless, this was not the case with an equally low neuronal differentiation in the undifferentiated and differentiated cell groups. This is in stark contrast to other reports where pre-differentiated cells exhibited good survival with an improvement in the survival of DARPP-32 cells [9,18,49]. There is indeed evidence that pre-differentiation of cells makes them especially vulnerable to apoptosis [50]. Improving overall cell survival might therefore also potentially increase the survival of pre-differentiated cells, but as in Parkinson’s disease additional survival factors (e.g. BDNF, GDNF) might be required to ensure the long-term survival and integration of these neurons [51,52].

Apart from poor cell survival and differentiation, it is plausible that, even if these issues are overcome, this cell line is not efficacious in Huntington’s disease. If this would indeed be the case, this cell line would provide an indispensable “therapeutic control” condition against which mechanisms of efficacious cells could be compared. Nevertheless, it is also conceivable that this cell line could provide efficacious results if implanted under different experimental conditions.

### Choosing an appropriate animal model of Huntington’s disease

STROC05 cells might be efficacious for Huntington’s disease, but it is possible that testing them in the R6/2 model does not reflect their therapeutic potential. The R6/2 model rapidly manifests behavioural impairments, as well as regional brain atrophy. This rapid progression of disease might be appropriate for screening pharmacological agents that exert immediate effects, but the time window might be too short and aggressive to evaluate the efficacy of neural progenitor/stem cells. Mouse models that develop neuronal loss over a protracted time course, such as the YAC72 [53] or HDH^(CAG)150^[54] might hence provide more appropriate conditions to establish the therapeutic efficacy of intracerebral cell implantation. Neural progenitor/stem cell implantation typically takes several weeks before therapeutic effects are evident. Therefore when R6/2 mice are almost moribund, implanted cells are expected to exert their effect and the disease might have progressed too far at this stage for any efficacy to be apparent. Additionally, the progression of the disease could impact on the cell’s survival [55]. Similar observations were evident in a previous study in R6/2 mice using fetal primary tissue grafts, where there was sufficient graft survival, but no meaningful therapeutic efficacy [56], although the same type of graft provided a significant improvement in neurotoxin-induced lesions modelling Huntington’s disease [13]. A similar difference in behavioural recovery between neurotoxic lesions in the mouse and the R6/2 were also observed after an intrastriatal injection of mesenchymal stem cells [57]. Merely implanting neural progenitor/stem cells in transgenic mice might hence be insufficient to achieve therapeutic efficacy.

A combination of treatments for various aspects of the disease might be needed for implanted cells to be efficacious. For instance, an injection of only mouse neural progenitors did not maintain motor function in N171-82Q transgenic mice, but if these same progenitors were engineered to also secrete GDNF, they provided a therapeutic benefit [58]. In the R6/2 mice, therapeutic efficacy was also achieved with NSCs, but only if these were administered in conjunction with a retardation of CAG aggregate formation using trehalose [59]. Transgenic mice therefore are likely to be appropriate models for establishing therapeutic efficacy in Huntington’s disease, but a combinatorial approach that concurrently impacts on different disease mechanisms might be needed to progress cell implantation as a treatment strategy. Having to target multiple mechanisms of the disease, as well as supplying novel cells to the brain, are likely to be a better reflection of the clinical condition than expecting neural progenitor/stem cells to be sufficiently efficacious to avert a further deterioration of patients.

## Conclusions

Neither the implantation of undifferentiated, nor pre-differentiated human NSCs promoted behavioural benefits or attenuated the on-going neurodegenerative process. This is likely due to a combination of factors, most importantly cell survival was insufficient to impact on the progression of the disease, but the life-span of R6/2 mice might also be too short to appropriately evaluate neural progenitors/stem cells. More chronic transgenic models are likely to be better in evaluating these therapies. However, implantation of cells by themselves is unlikely to be sufficiently efficacious to promote recovery, but rather a combination of multiple treatments will be required to provide a truly efficacious therapy that can impact on the clinical condition.

## Competing interests

MM previously received financial and personnel support from ReNeuron Ltd to study the efficacy of a hNSC line in a rat model of stroke.

## Authors’ contributions

GEA: design of study, in vitro experiments, behavioural testing, magnetic resonance imaging, stereotactic surgery, post-mortem histology, statistical analyses, first draft of manuscript; IR: design of study, stereotactic surgery, magnetic resonance imaging; revising of manuscript; SG: conducted the 3-NPA study; RG: breeding and genotyping of animals; GB: conception of study, financial support, revising of manuscript; MM: conception and design of study; financial support; statistical analyses; drafting of manuscript; final approval of manuscript. All authors read and approved the final manuscript.

## Supplementary Material

Additional file 1 **Figure S1.** STROC05 survival in the 3-nitroproprionic acid (3-NPA) rat model of Huntington’s disease. Male Lewis rats (220-250 g) received i.p. injections of 42 mg/kg 3-NPA. (Sigma-Aldrich) for five consecutive days to induce a bilateral degeneration of striatal cells, as previously described [8]. Animals gradually develop a behavioural phenotype and show a progressive striatal tissue loss that coincides with neuronal loss, as well as an increase in glial scarring and microglia activity [8]. Additionally, these animals show a clear deficit in brain activity [7,60]. Two weeks after lesion induction, animals received unilateral injections of 400,000 STROC05 human neural stem cells (hNSCs). hNSCs can be detected in the injection tract using human nuclear antigen (A). The presence of CD11b + microglia reveals the inflammatory response to the ongoing neurodegeneration in the lateral striatum and indicates a placement of cells just peripheral to the damage. Higher magnification images reveal a limited migration from the injection tract to the area of damage (B&C). STROC05 cells retained some expression of nestin (D&E), but also partially differentiated into GFAP + astrocytes. Using brightfield microscopy of cell survival (G) in animals that were either immunocompetent or immunosuppressed using Cyclosporine A (CsA, Sandimmun, Novartis, 10 mg/kg, diluted in Ringer’s solution) and methylpredinolone (20 mg/kg day 1–7; 10 mg/kg day 8–12; 5 mg/kg day 13–14 i.p., Pharmacia Upjohn), a sterelogical analysis indicated a robust cell survival under both conditions over 90 days. Over 10,000 cells survived in the immunocompetent group and 25,000 cells were present in the immunosuppressed rats. It was only at 90 days survival that there was a significant difference between immunosuppression and immunocompetent animals (* P < .05), but there was no significant decrease in cell number between 30 and 90 days. Discontinuation of immunosuppression also did not lead to a graft rejection. Click here for file

Additional file 2 **Figure S2.** Acute survival of STROC05 in WT mice. An injection of 225,000 STROC05 cells in 3 μl (75,000 cells/μl) at 7 weeks of age into wild-type mice resulted in a good graft survival (Human nuclei antigen, HNA, in red, DAPI in blue), even in the absence of immunosuppression. Cells remained within the injection tract and did not exhibit any migration out of their site of injection. To ensure a better distribution of cells within the striatum, two deposits were placed within the same injection tract. A glial reaction (GFAP + cells in green) was evident along the injection tract. These results indicate that STROC05 cells can survive in WT animals and that using this protocol there is a robust engraftment. Click here for file

Additional file 3 **Figure S3.** Representative T_2_x-weighted MRI images. Images illustrated the anatomical boundaries used to define regions of interests corresponding to anatomical structures (red lines). Click here for file

Additional file 4 **Figure S4.** Re-analysis of the main outcome measures. As some animals had no graft survival, it is conceivable that this would affect the group outcome measure. Therefore we reanalysed the data excluding these animals. The analysis containing all animals is presented on the left and the reanalysed data on the right. Exclusion of animals without graft survival, however, did not make a difference to these results. Click here for file
