# Supplementary figures and images for: Implantation of undifferentiated and pre-differentiated human neural stem cells in the R6/2 transgenic mouse model of Huntington’s disease
Source: BMC Neurosci. 2012 Aug 9;13:97. doi: 10.1186/1471-2202-13-97 (PMC3502570; doi:10.1186/1471-2202-13-97)

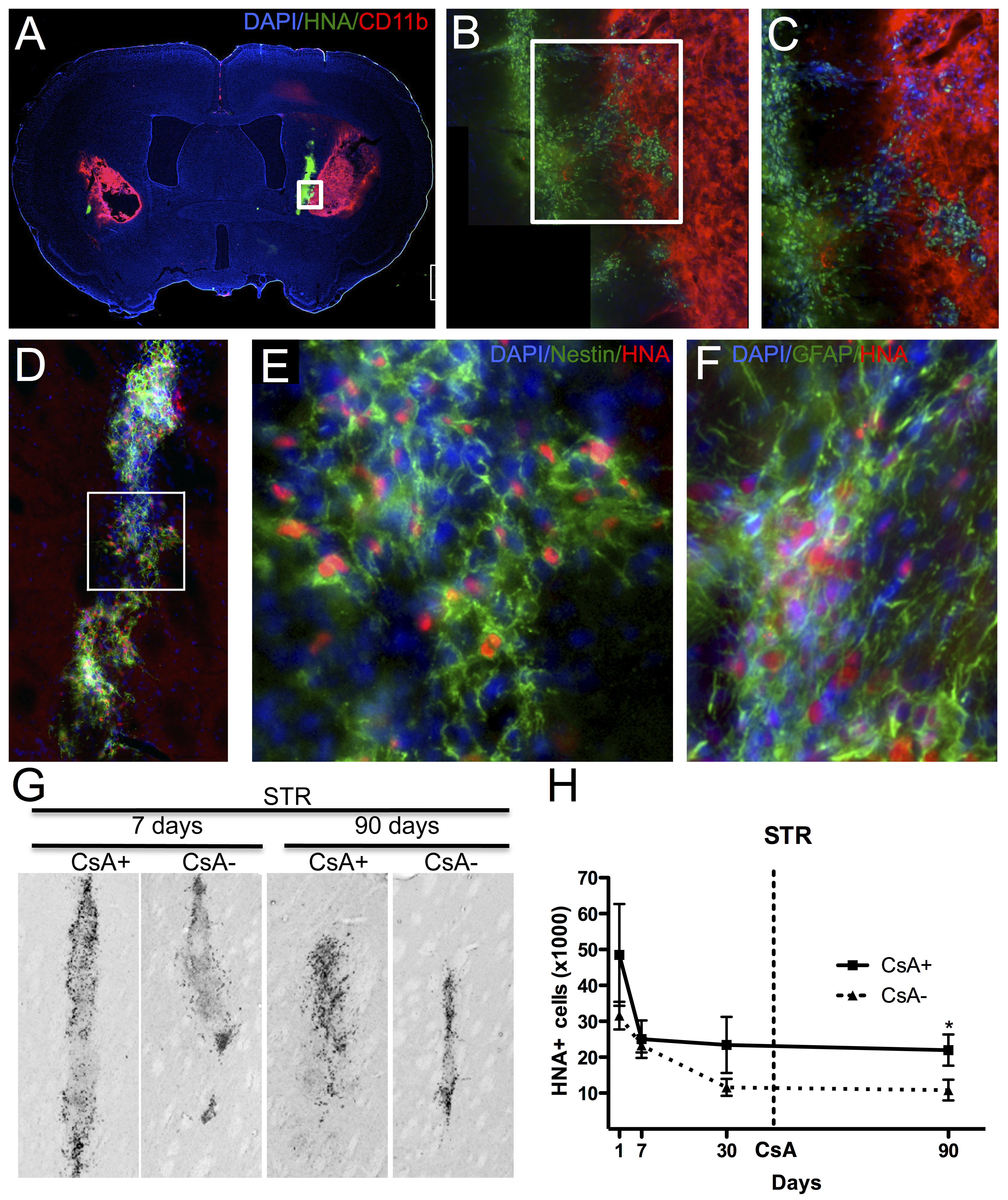

Supplement: Additional file 1 — Figure S1. STROC05 survival in the 3-nitroproprionic acid (3-NPA) rat model of Huntington’s disease. Male Lewis rats (220-250 g) received i.p. injections of 42 mg/kg 3-NPA. (Sigma-Aldrich) for five consecutive days to induce a bilateral degeneration of striatal cells, as previously described [8]. Animals gradually develop a behavioural phenotype and show a progressive striatal tissue loss that coincides with neuronal loss, as well as an increase in glial scarring and microglia activity [8]. Additionally, these animals show a clear deficit in brain activity [7,60]. Two weeks after lesion induction, animals received unilateral injections of 400,000 STROC05 human neural stem cells (hNSCs). hNSCs can be detected in the injection tract using human nuclear antigen (A). The presence of CD11b + microglia reveals the inflammatory response to the ongoing neurodegeneration in the lateral striatum and indicates a placement of cells just peripheral to the damage. Higher magnification images reveal a limited migration from the injection tract to the area of damage (B&C). STROC05 cells retained some expression of nestin (D&E), but also partially differentiated into GFAP + astrocytes. Using brightfield microscopy of cell survival (G) in animals that were either immunocompetent or immunosuppressed using Cyclosporine A (CsA, Sandimmun, Novartis, 10 mg/kg, diluted in Ringer’s solution) and methylpredinolone (20 mg/kg day 1–7; 10 mg/kg day 8–12; 5 mg/kg day 13–14 i.p., Pharmacia Upjohn), a sterelogical analysis indicated a robust cell survival under both conditions over 90 days. Over 10,000 cells survived in the immunocompetent group and 25,000 cells were present in the immunosuppressed rats. It was only at 90 days survival that there was a significant difference between immunosuppression and immunocompetent animals (* P < .05), but there was no significant decrease in cell number between 30 and 90 days. Discontinuation of immunosuppression also did not lead to a graft rejection. [file 1471-2202-13-97-S1.tiff]

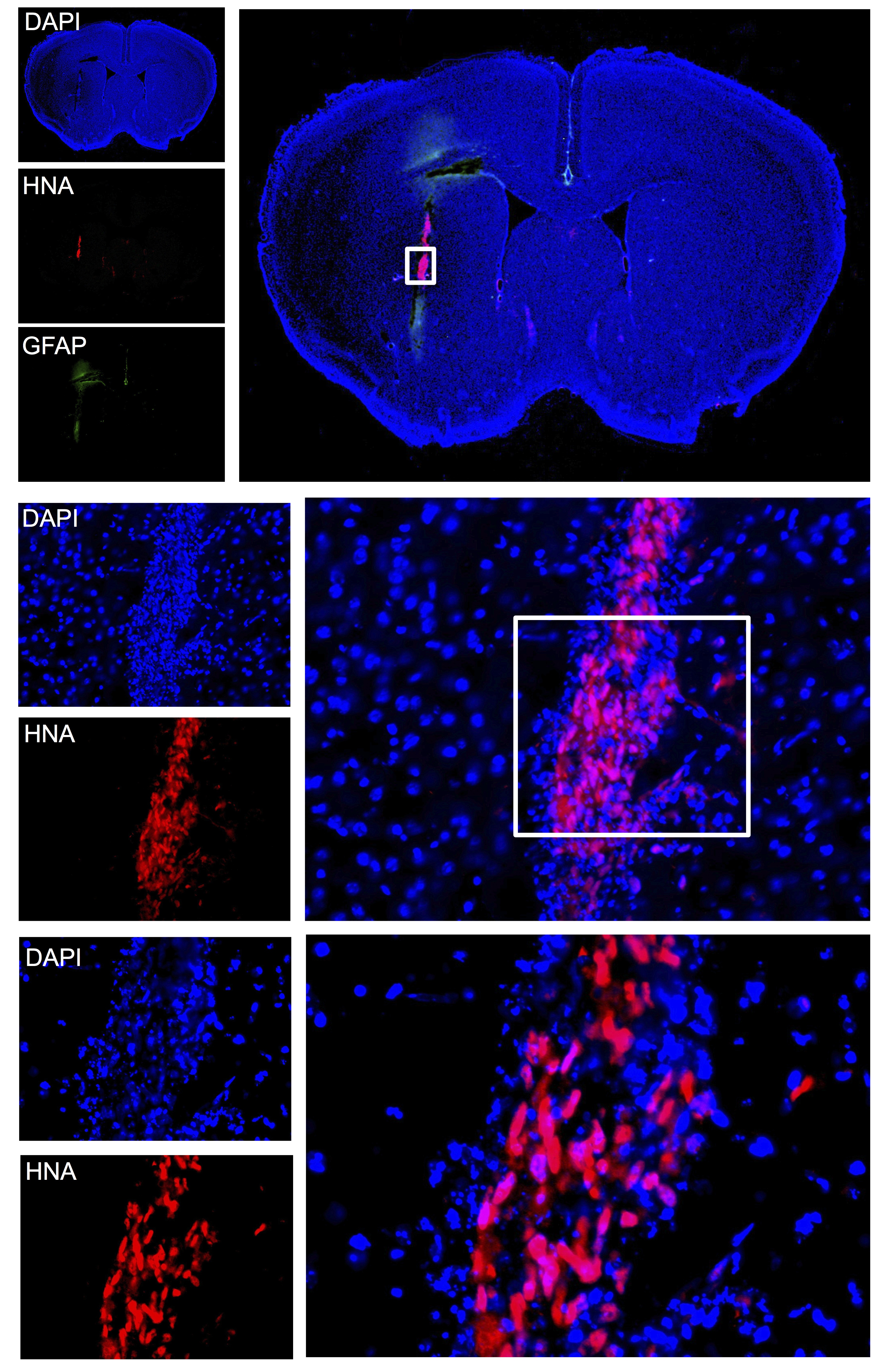

Supplement: Additional file 2 — Figure S2. Acute survival of STROC05 in WT mice. An injection of 225,000 STROC05 cells in 3 μl (75,000 cells/μl) at 7 weeks of age into wild-type mice resulted in a good graft survival (Human nuclei antigen, HNA, in red, DAPI in blue), even in the absence of immunosuppression. Cells remained within the injection tract and did not exhibit any migration out of their site of injection. To ensure a better distribution of cells within the striatum, two deposits were placed within the same injection tract. A glial reaction (GFAP + cells in green) was evident along the injection tract. These results indicate that STROC05 cells can survive in WT animals and that using this protocol there is a robust engraftment. [file 1471-2202-13-97-S2.jpeg]

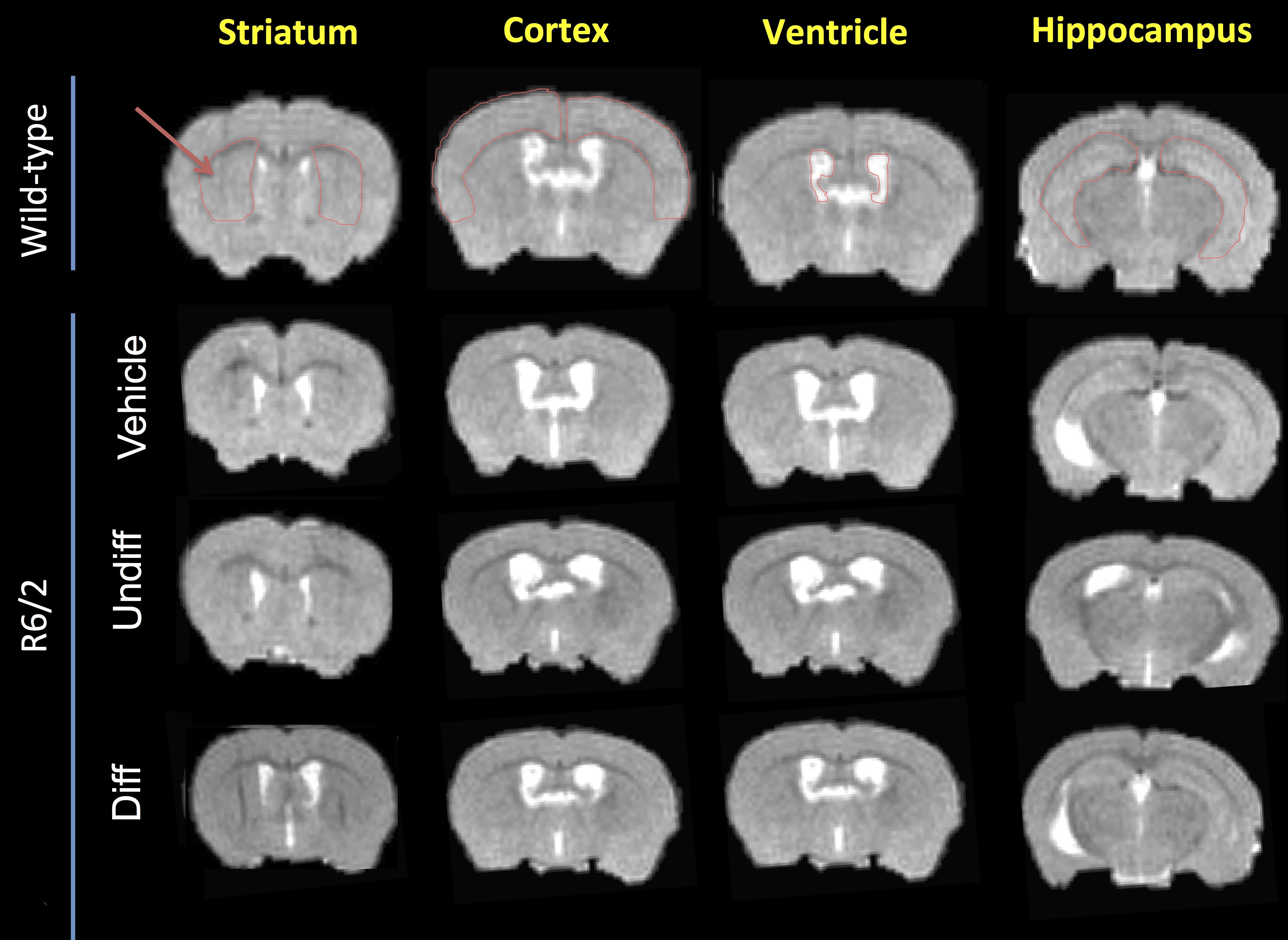

Supplement: Additional file 3 — Figure S3. Representative T2x-weighted MRI images. Images illustrated the anatomical boundaries used to define regions of interests corresponding to anatomical structures (red lines). [file 1471-2202-13-97-S3.tiff]

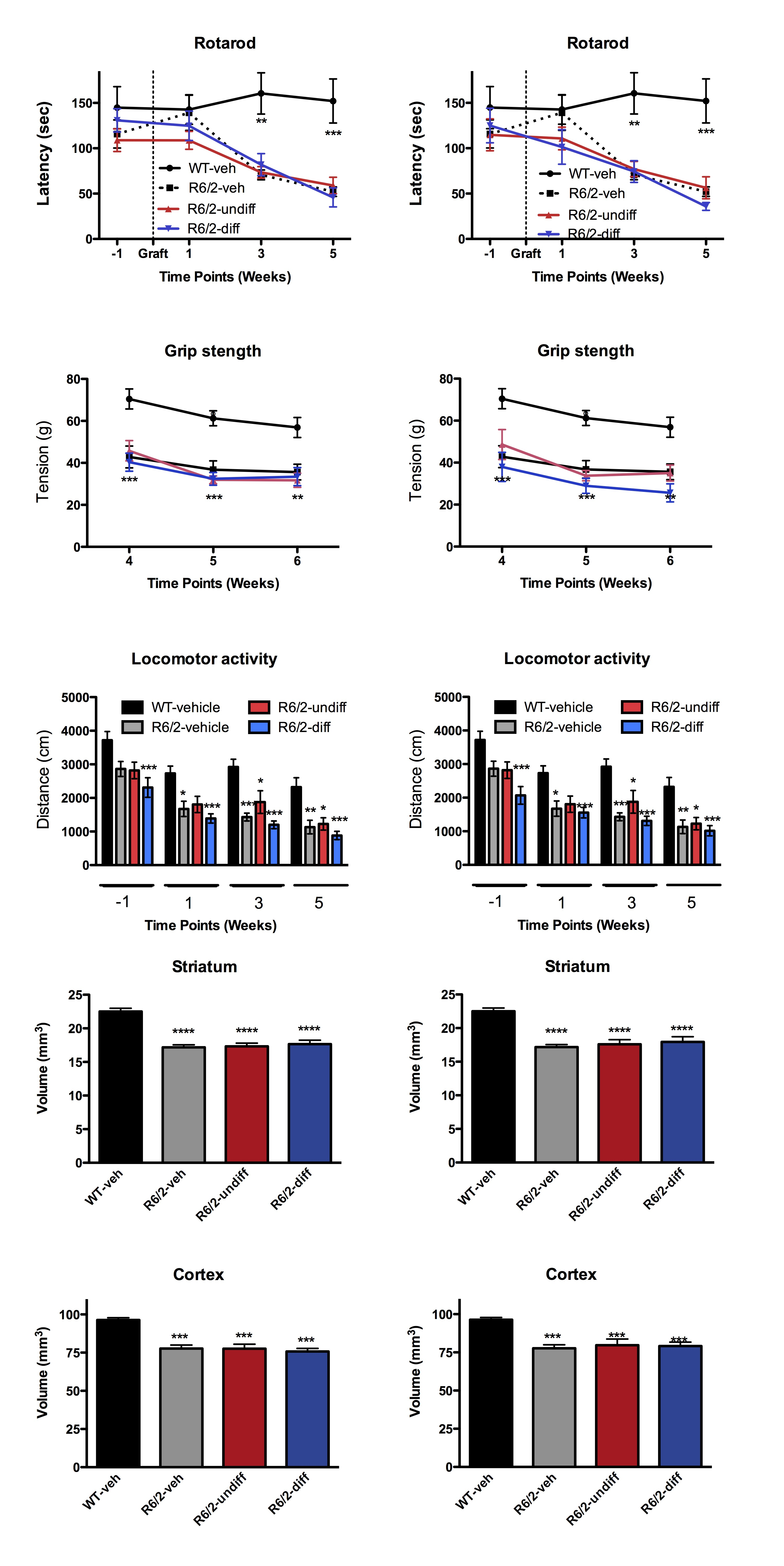

Supplement: Additional file 4 — Figure S4. Re-analysis of the main outcome measures. As some animals had no graft survival, it is conceivable that this would affect the group outcome measure. Therefore we reanalysed the data excluding these animals. The analysis containing all animals is presented on the left and the reanalysed data on the right. Exclusion of animals without graft survival, however, did not make a difference to these results. [file 1471-2202-13-97-S4.tiff]
